# Gallbladder Pancreatic Heterotopia—The Importance of Diagnostic Imaging in Managing Intraoperative Findings

**DOI:** 10.3390/medicina59081407

**Published:** 2023-08-01

**Authors:** Crenguţa Sorina Şerboiu, Cătălin Aliuș, Adrian Dumitru, Dana Țăpoi, Mariana Costache, Adriana Elena Nica, Mihăilescu Alexandra-Ana, Iulian Antoniac, Sebastian Grădinaru

**Affiliations:** 1 Department of Cellular, Molecular Biology and Histology, Carol Davila University of Medicine and Pharmacy, 050474 Bucharest, Romania; crengutas@yahoo.com; 2 Department of Radiology and Medical Imaging, University Emergency Hospital Bucharest, 050098 Bucharest, Romania; 3 Surgical Department IV, University Emergency Hospital Bucharest, 050098 Bucharest, Romania; 4 Pathology Department, University Emergency Hospital Bucharest, 050098 Bucharest, Romania; dr.adriandumitru@yahoo.com (A.D.); dana-antonia.tapoi@drd.umfcd.ro (D.Ț.); m_costache_dermatopat@yahoo.com (M.C.); 5 Intensive Care Unit, University Emergency Hospital Bucharest, 050098 Bucharest, Romania; adriana.nica@suub.ro; 6 Intensive Care Unit, Foisor Hospital Bucharest, 021382 Bucharest, Romania; mihailescu.alexandra@drd.umfcd.ro; 7 Department of Metallic Materials Sciense and Physical Metallurgy, Faculty of Materials Science and Engineering, University Politehnica of Bucharest, 060042 Bucharest, Romania; antoniac.iulian@gmail.com; 8 Department of General Surgery, County Hospital Ilfov, 050474 Bucharest, Romania; gradinarusebastian@gmail.com; 9 Titu Maiorescu University of Medicine and Pharmacy, 031593 Bucharest, Romania

**Keywords:** ectopic, pancreas, heterotopia, gallbladder, pancreatitis, cholecystitis

## Abstract

Pancreatic heterotopy is a rare entity defined as the presence of abnormally located pancreatic tissue without any anatomical or vascular connection to the normal pancreas. Heterotopic pancreatic tissue can be found in various regions of the digestive system, such as the stomach, duodenum, and upper jejunum, with the less commonly reported location being the gallbladder. Gallbladder pancreatic heterotopia can be either an incidental finding or diagnosed in association with cholecystitis. Pancreatitis of the ectopic tissue has also been described. In this context, we report three cases of heterotopic pancreatic tissue in the gallbladder with different types of pancreatic tissue according to the Heinrich classification. One patient was a 24-year-old male who presented with acute pancreatitis symptoms and an ultrasonographical detected mass in the gallbladder, which proved to be heterotopic pancreatic tissue. The other two cases were female patients aged 24 and 32, respectively, incidentally diagnosed on histopathological examination after cholecystectomy for symptomatic cholelithiasis. Both cases displayed chronic cholecystitis lesions; one of them was also associated with low grade dysplasia of the gallbladder. Although a rare occurrence in general, pancreatic heterotopia should be acknowledged as a possible incidental finding in asymptomatic patients as well as a cause for acute cholecystitis or pancreatitis.

## 1. Introduction

A heterotopic pancreas is described as a pancreatic tissue that does not have any anatomical or vascular connections with the pancreas itself. Other terms such as “ectopic pancreas”, “accessory pancreatic tissue”, “pancreatic choristoma”, or “aberrant pancreas” are used in the literature. It is most commonly found in the gastrointestinal tract, with over 90% of the cases reported in its upper part (stomach, duodenum, and upper jejunum). Some rare locations that were reported in association with pancreatic heterotopia include the ileum, Meckel’s diverticulum, splenic hilum, common bile duct, and in exceptionally rare cases, the gallbladder [1,2,3]. Despite being a congenital anomaly pancreatic heterotopia is usually an incidental finding, reported in 13% of cases at autopsy and in only 0.2% after laparotomy [4,5]. Although the majority of these pancreatic remnants are functional, they are usually asymptomatic [6]. When symptoms arise, they are mainly non-specific and consist of abdominal pain or tenderness, dyspepsia, nausea, vomiting, anaemia, or gastrointestinal bleeding [4,7]. Pancreatitis of the ectopic tissue has also been described [8]. In this paper, we report three cases of gallbladder heterotopic pancreas, an entity so rare that there have been fewer than 40 cases reported since 1916 [4]. As highlighted in similar studies reporting uncommon conditions, awareness and dissemination are crucial in helping clinicians ensure early diagnosis and appropriate management. Furthermore, advancements in histopathological analysis, immunohistochemistry techniques, and the introduction of artificial intelligence in specimen readings are expected to increase the number of papers on rare findings.

**Figure 1:**
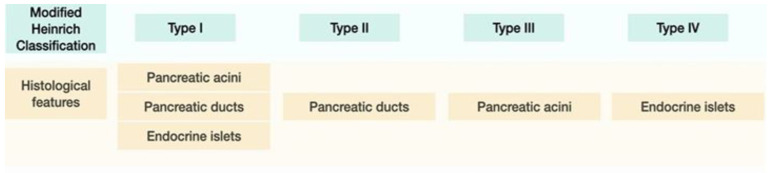
Modified Heinrich Classification for pancreatic heterotopia.

The first histological classification of pancreatic heterotopia in the gallbladder was published by von Heinrich more than a hundred years ago [9] and was last modified by Gaspar Fuentes et al. in the early 1970s [10]. According to this classification, there are four types of pancreatic heterotopia (Figure 1).

Type I displays pancreatic acini, ducts, and endocrine islets; type II displays pancreatic ducts only; type III displays acini only; and type IV endocrine cells only. Although acini and ducts are visible in conventional HE staining, immunohistochemistry can be employed to identify and confirm the presence of acinar/ductal components using Cytokeratin. The endocrine islets could be identified based on the presence of neuroendocrine markers such as Chromogranin A (non-specific) and Insulin (specific).

## 2. Types of Pancreatic Heterotopia

### 2.1. Type 1

**Figure 2:**
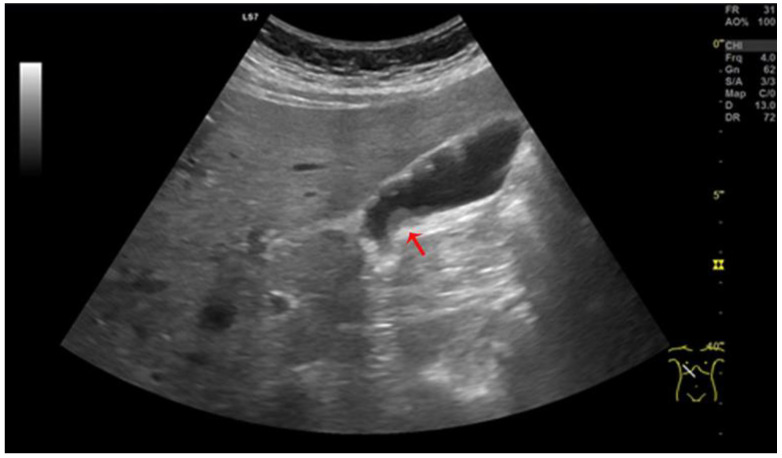
Ultrasonography reveals pancreatic heterotopia Type 1 (red arrow).

A 24-year-old man presented to the hospital with intense upper abdominal pain radiating to the back, nausea, and vomiting. The patient requested medical help after excessive alcohol consumption, so the initial supposition was acute pancreatitis. On physical examination, the abdomen was tender in the right upper quadrant with a positive Murphy’s sign. The white cell count was normal but with a left shift of the leukocytes and normal amylase and lipase levels. Ultrasonography revealed a mass initially believed to be an enlarged Mascagni’s lymph node, and therefore laparoscopic cholecystectomy was recommended (Figure 2). Upper GI endoscopy revealed no signs of peptic ulcer disease and redirected the diagnosis toward biliary pathology since the only objective findings were a thickened gallbladder wall and an enlarged juxta cystic node. A laparoscopic cholecystectomy was indicated, and intraoperative findings showed a globulous gallbladder with an edematous wall and pericholecystic adhesions. At the time of the operation, a diagnosis of acute cholecystitis was supported by the clinical presentation and the oedematous walls of the gallbladder. The postoperative course of the patient was favorable, with remission of the symptoms and discharge after three days.

**Figure 3:**
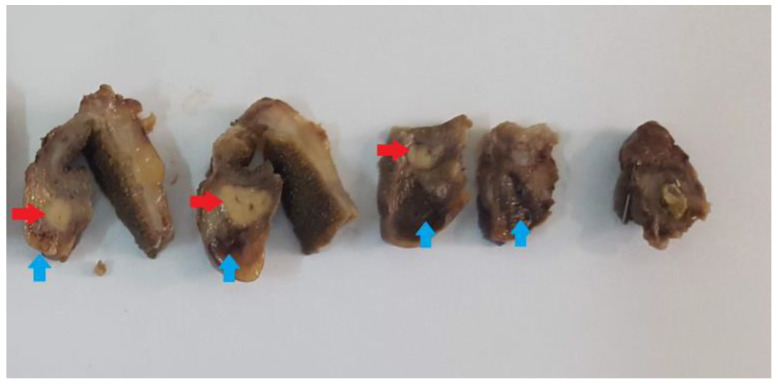
Gross appearance of the heterotopic pancreatic tissue in the wall of the gallbladder (red arrow). Note the Mascagni lymph node in the vicinity (blue arrow).

After the surgical removal of the gallbladder, the tissue fragments were sent for processing and examination to the Department of Pathology. Gross examination revealed a slightly lobulated white-yellowish pseudo-tumoral mass measuring approximately 1.8/2/1.2 cm (Figure 3). The mass was found in the infundibular area of the gallbladder, adjacent to Mascagni’s lymph node. Tissue samples were fixed with 10% buffered Formalin and sent for histopathological processing by conventional methods using Paraffin inclusion and Hematoxylin-Eosin (HE) staining. Also, immunohistochemical tests were performed. The paraffin blocks acquired by histopathological processing were sectioned at the microtome resulting in 2 μm thickness sections mounted on slides covered with poly-L-Lysin. We used the following antibodies from Biocare for ancillary testing: Cytokeratin 8/18, Cytokeratin 19, Chromogranin A, and Insulin. Cytokeratin (Ck) has been used to highlight serous acini and /or pancreatic ducts, while Langerhans cells have been highlighted using either a universal marker for neuroendocrine differentiation (Chromogranin A) or a specific immunomarker: Insulin.

**Figure 4:**
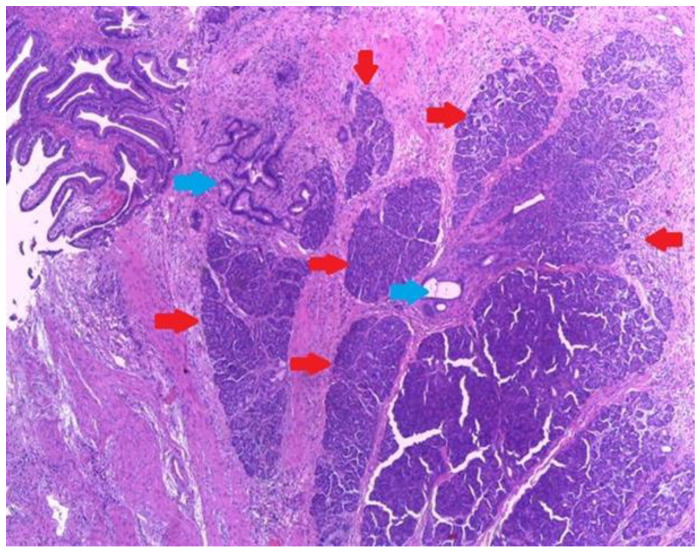
Heterotopic pancreatic tissue of the gallbladder. Hematoxylin and Eosin staining, 4× magnification. It can be observed in serous acini (red arrows) and exocrine ducts (blue arrows).

**Figure 5:**
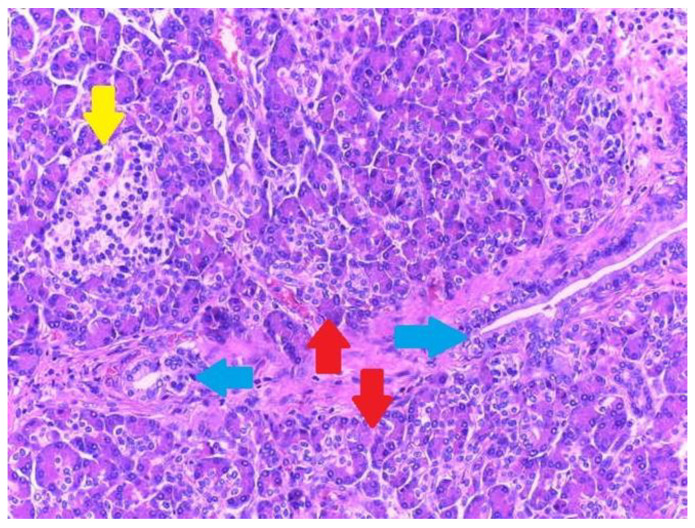
Type 1 gallbladder pancreatic heterotopia according the de modified Heinrich classification. All three histological elements of the pancreas can be observed: serous acini (red arrows), endocrine cells (yellow arrow) and exocrine ducts (blue arrow).

**Figure 6:**
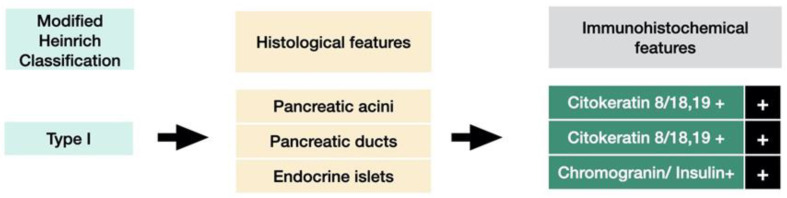
Histological features and IHC (imunohistochemistry) features of type I pancreatic heterotopia.

Microscopic examination showed a well-circumscribed fully developed heterotopic pancreatic tissue in the muscularis layer extending to the subserosa adipose connective tissue. The pancreatic rests were composed of lobules of exocrine pancreatic acini, exocrine ducts and many Langerhans islets. (Figure 4 and Figure 5). The findings were consistent with type I pancreatic heterotopia according to the modified Heinrich classification (Figure 6).

**Figure 7:**
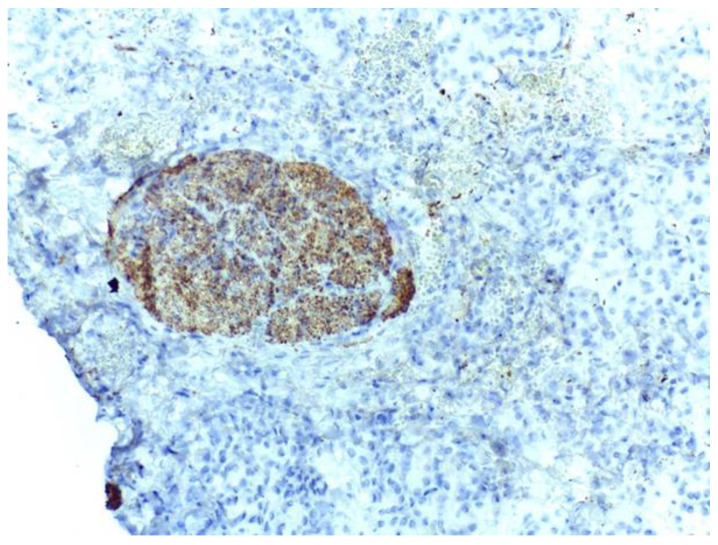
Langerhans islets are strongly positive for Insulin antibodies in heterotopic pancreatic tissue of the gallbladder. IHC staining with DAB chromogen, 20× magnification.

**Figure 8:**
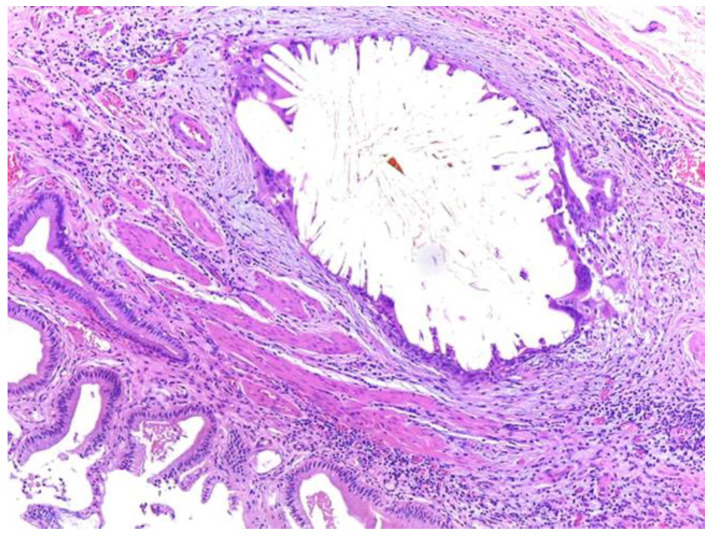
Microscopic aspects suggestive of chronic cholecystitis. Observe a cholesterol calculus included in a Rokitansky-Aschoff sinus. Hematoxylin and Eosin staining, 10× magnification.

The islets were strongly and diffusely positive for Chromogranin A and Insulin (Figure 7). The serous acini were positive for Ck8/18. The remaining sections showed features of chronic cholecystitis (Figure 8).

The discovery of the heterotopic tissue was incidental, the emergency surgery enabled the removal of the gallbladder with subsequent remission of the symptoms. The patient presented follow-up without any further pathology.

### 2.2. Type 2

**Figure 9:**
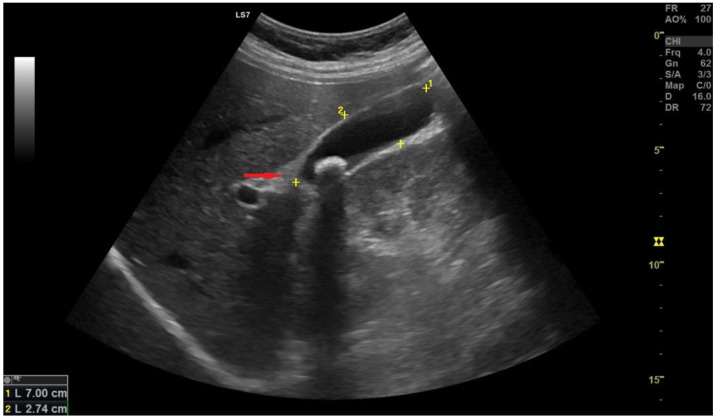
Ultrasonography reveals the site of pancreatic ectopy type 2 (red arrow), opposite the macro calculus with posterior shadow cone.

A 24-year-old female presented to the hospital with a history of right hypochondriac pain, nausea, nausea and vomiting. No change in the hematology or biochemistry panels, was noted. On physical examination, there was tenderness in the right upper quadrant of the abdomen. Abdominal ultrasound examination showed cholelithiasis, and the patient underwent laparoscopic cholecystectomy, (Figure 9). The intraoperative findings showed a thickened gallbladder wall and unremarkable neighboring viscera. The postoperative course was uneventful, and the patient was discharged home after two days free of symptoms. Follow-up with the general practitioner was uneventful, and no further visits were requested for this patient. Macroscopic examination did not reveal particular aspects other than those suggestive of chronic cholecystitis. On microscopic examination, ectopic pancreatic tissue was and an incidental finding, being observed within the gallbladder wall.

**Figure 10:**
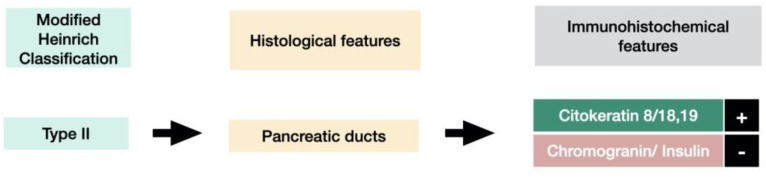
Histological features and IHC features of type II pancreatic heterotopia.

**Figure 11:**
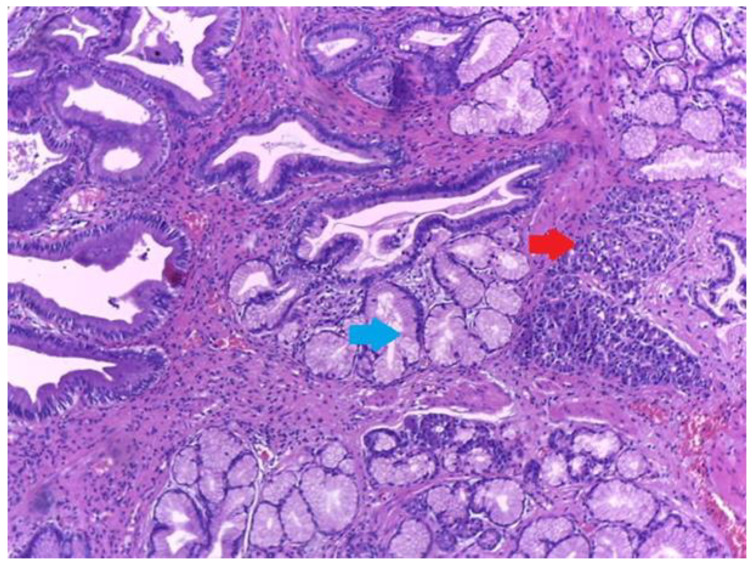
Small foci of heterotopic pancreatic tissue of the gallbladder (red arrow) in the vicinity of pseudo-pyloric metaplasia (blue arrow). Hematoxylin and Eosin staining, 10× magnification.

**Figure 12:**
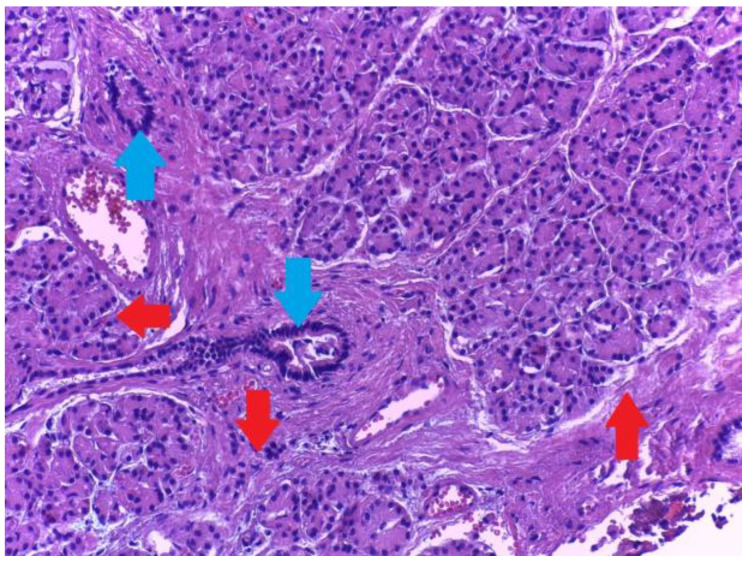
Small foci of heterotopic pancreatic tissue of the gallbladder (red arrow) in the vicinity of pseudo-pyloric metaplasia (blue arrow). High power magnification showing serous acini and exocrine ducts only in the heterotopic pancreatic tissue of the gallbladder (type 2 heterotopic pancreatic tissue according to the Heinrich classification). Hematoxylin and Eosin staining, 40× magnification.

The ectopic tissue was composed of serous acini and a few exocrine ducts but no endocrine islets, thus this case was classified as type 2 pancreatic heterotopia according to the modified Heinrich’s criteria (Figure 10). Pseudo-pyloric metaplasia was observed in the vicinity of the ectopic tissue (Figure 11 and Figure 12).

### 2.3. Type 3

**Figure 13:**
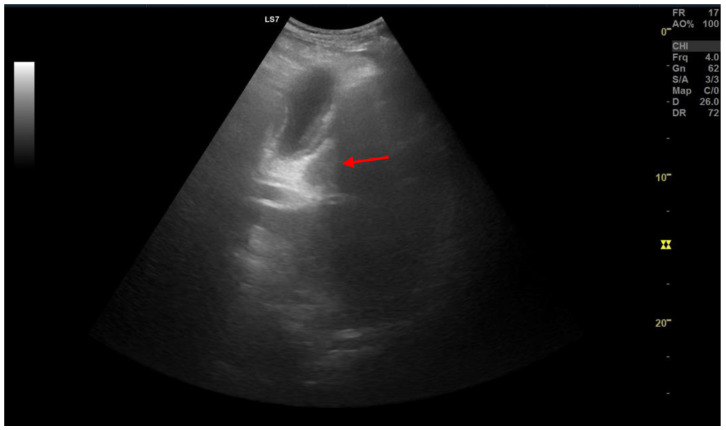
Ultrasonography of a case with type 3 pancreatic heterotopy (red arrow).

A 32-year-old female presented to the hospital with a history of right upper abdominal pain radiating to the right shoulder, nausea, and vomiting, particularly associated with fatty food intake. The patient had a history of neglected recurrent abdominal pain, and initially used over-the-counter analgesics for self-medication. The right hypochondriac region was tender on physical examination with no change in the CBC (Complete Blood Count) panel. Ultrasonography revealed cholelithiasis and subsequently laparoscopic cholecystectomy was carried out, (Figure 13). On gross examination variable thickening of the gallbladder wall was observed. The wall thickness varied from 0.3 to 0.6 cm. The serosal layer was unremarkable. The mucosa was flattened and focally ulcerated and numerous yellowish-green friable round stones, each measuring up to 0.4 cm in diameter, were noted in the fundus.

**Figure 14:**
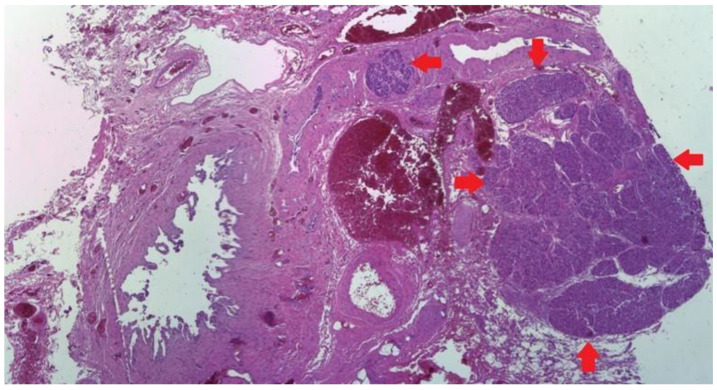
Heterotopic pancreatic tissue (serous acini only-red arrow) in the neck region of the gallbladder. Hematoxylin and Eosin staining, 4× magnification, Leica stitch image software resolution.

**Figure 15:**
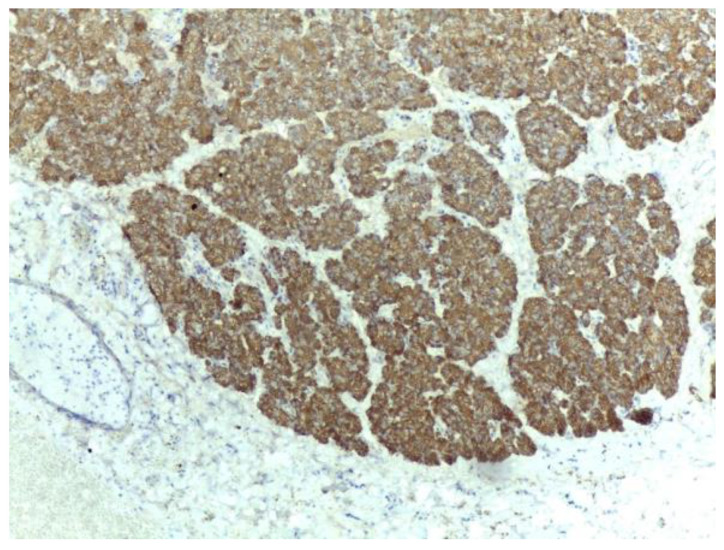
The serous pancreatic acini were strongly positive for Ck8/18 antibody. IHC staining with DAB (Diaminobenzidine) chromogen, 10× magnification.

**Figure 16:**
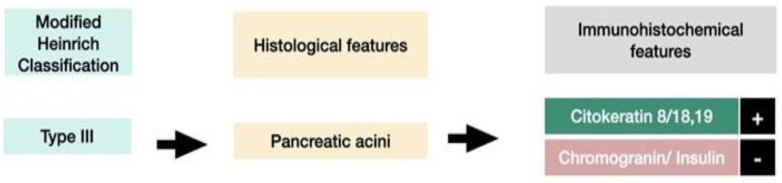
Histological features and IHC features of type I pancreatic heterotopia.

**Figure 17:**
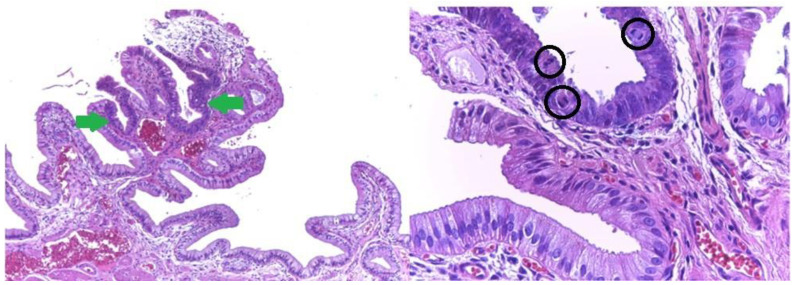
Low grade biliary intraepithelial neoplasia (BilIN1): flat architecture, basal nuclei, pseudo-stratification within lower two thirds of epithelium (green arrows). Note the mild nuclear abnormalities and frequent mitotic figures (black circles). Hematoxylin and Eosin staining, 10× and 40× magnifications.

A grey-tanned area was seen in the neck region, thought to be the Mascagni’s lymph-node, which, instead, on microscopic examination, showed to be a well-circumscribed rest of heterotopic pancreatic tissue, composed of tightly packed lobules of exocrine pancreatic acini (Figure 14). Exocrine ducts or Islets of Langerhans were not seen. The serous acini were strongly positive for Ck 8/18 (Figure 15). This type of heterotopy corresponds to type 3 after Gaspar-Fuentes classification (Figure 16), the original Heinrich classification not having this subtype. The remaining sections showed features of chronic cholecystitis but the incidental findings did not stop here: the mucosa had focal aspects of low -grade biliary intraepithelial dysplasia (Figure 17).

## 3. Discussion

Heterotopic pancreas is a congenital anomaly defined as the presence of pancreatic tissue abnormally located without any connection to the normal pancreas itself [11]. Heterotopic pancreas is usually located in the stomach, small intestine and Meckel’s diverticulum [1,2,3] and it has been rarely encountered in other sites such as the liver or spleen [12]. Gallbladder pancreatic heterotopia was first described by Otschkin in 1916 [13,14] and to this date there are less than 40 cases reported in the literature [15]. It is most frequently located close to the neck of the gallbladder [16]. The overall incidence of pancreatic heterotopia is estimated to be up to 13% on autopsy and 0.2% after laparotomy [4,5]. All these data reflect a low prevalence of heterotopia but suggest ubiquitous potential localizations.

Since the ectopic pancreas is affected by the same pathologies as the orthotopic tissue the clinician should expect pancreatitis, pseudocyst formation, bleeding and malignant transformation hence alterations of the classical clinical patterns would be a natural occurrence. In a young patient such as our first case the presentation with acute upper abdominal pain radiating to the back was suggestive of acute pancreatitis. An elevation of the pancreatic enzymes (although notthree to five times higher than the normal value) would fulfil two Atlanta criteria for acute pancreatitis. In the absence of clinical deterioration repeat imaging in pancreatitis is usually performed three days after the onset of the symptoms to allow for mounting of pathological changes visible on USS or CT. On the other hand, the latest Tokyo criteria for the management of acute pancreatitis indicate that for grade I and II acute cholecystitis, early cholecystectomy should be performed within the first 72 h of admission. But this case had only a partially thickened gallbladder wall with no obvious oedematous changes. The USS appearance of the ectopic pancreas is usually an echogenic mass of variable dimensions located mainly near the cystic duct or in the neck of the gallbladder, hence the potential confusion with a lymph node. From acute pancreatitis to dyspepsia and peptic ulcer disease the spectrum of conditions causing similar clinical presentations is large therefore we believe that pancreatic heterotopia, although rare, is a condition that must be considered for differential diagnoses, especially in cases with borderline criteria for acute pancreatitis or lack of correlation between clinical features and imaging findings. The second case presented was a routine case of symptomatic cholelithiasis having atypia diagnosed incidentally on the histology report. There is no data in the literature to suggest a correlation between the presence of pancreatic heterotopia and gallbladder lithiasis. In addition to this, the asymptomatic proportion of patients with both these conditions is quite high. A study performed by Wei on patients with upper gi pancreatic heterotopia suggested that up to 50 per cent of the patients are symptomatic, but because of its low prevalence and the superposable symptoms of more common conditions, underdiagnosis is very likely. The third case was another incidental finding but with importance due to the microscopic changes suggestive of a different type of pancreatic heterotopia. The rarity of these cases coupled with underdiagnosis makes the topic relevant for the clinician who deals with gastrointestinal conditions, especially in acute cases. The occurrence of all three types of pancreatic heterotopia in a single centre while less than 40 cases of gallbladder localisation were reported so far makes the allegation of underdiagnosis even more apt and suggests that perhaps this diagnosis should be sought more frequently.

Since pancreatic heterotopia is a result of abnormal embryological development there are a couple of theories about the mechanisms behind this erratic development. One theory claims that the heterotopic tissue is detached from the primitive pancreas during its rotation, while another theory alleges that during the development of the pancreatic bud, fragments of pancreatic tissue are separated by the longitudinal growth of the intestines [17,18,19]. Various other studies argue that abnormalities in the Notch signaling pathway may lead to the development of pancreatic heterotopia [20,21]. Hes-1 (Hairy enhancer of split) is a main effector of Notch system and it is involved in region-appropriate differentiation of the pancreas in the developing foregut endoderm. In this respect, the occurrence of pancreatic heterotopia has been noted in Hes-1 knockout mice [22].

**Figure 18:**
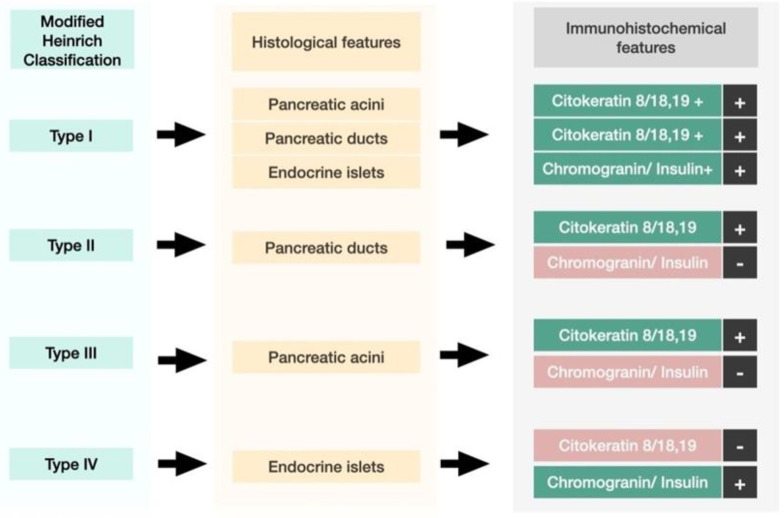
Histological features and IHC features of pancreatic atypia.

Histologically, heterotopic pancreas is composed of endocrine and exocrine tissue in various amounts. The first histological classification on pancreatic heterotopia was published by von Heinrich in 1909 and was lastly modified by Gaspar Fuentes et al. in 1973 [9]. According to this classification, there are 4 types of pancreatic heterotopia: Type I: acini, ducts, and islets of endocrine glands, Type II: canalicular variant with pancreatic ducts, Type III: exocrine pancreas with acinar tissue only. No endocrine tissue is present, Type IV: endocrine pancreas with cellular islets only (no exocrine tissue is present) (Figure 18).

In terms of clinical manifestations, pancreatic heterotopia is usually asymptomatic. When symptoms do arise, they are mostly a result of complications such as cholecystitis, bile duct and intestinal obstructions or mucosal ulcers [3], and even gallbladder perforation and peritonitis [18]. There are, however cases, when symptoms are related to the heterotopic pancreas itself. Although rare, pancreatic heterotopia in the stomach can undergo malignant transformation to pancreatic adenocarcinoma [23]. Tumors arising from heterotopic endocrine pancreatic tissue have also been described [24]. In addition to this, heterotopic pancreatic tissue can also cause acute and chronic pancreatitis [25]. Symptoms related to gallbladder pancreatic heterotopia most often resemble acute or chronic cholecystitis. Histopathological examination confirms both the existence of heterotopic pancreatic tissue and lesions of acute and chronic cholecystitis [4,6] It has been demonstrated that pancreatic enzymes levels are elevated in gallbladder bile and as a consequence could be a trigger for gallbladder lesion and therefore lead to acalculous cholecystitis and even gallbladder carcinoma [15,20]. Gallbladder pancreatic heterotopia with chronic cholecystitis was also found in association with pseudo-pyloric metaplasia, and adenomyomatous hyperplasia of the gallbladder [26].

In one of our own cases, the gallbladder mucosa displayed low grade dysplasia. Gallbladder surgery is the most common intervention worldwide with an increasing prevalence due to lifestyle modifications and the growth of obesity. Since every specimen must be sent for histology it is expected that the prevalence of pancreatic heterotopia to rise in correlation with the total number of cholecystectomies which approaches a million cases per year only in Europe. Despite these numbers, very few cases of heterotopia were reported so far. It is true that diagnosing and categorizing it requires the employment of immunohistochemistry techniques which are not universally available and are for some institutions too expensive for routine use.

Since rare diseases affect only a small number of people compared to the general population, this field suffers from a deficit of scientific and medical knowledge input. Hence scientific publications such as case reports and clinical trial studies represent a valuable resource helping to overcome the current imitations [27]. The complexity of this disease requires strong collaboration between the surgeon, radiologist and pathologist to be diagnosed and treated properly. Beside the disease complications, the intraoperative events are not to be neglected either [28].

## 4. Conclusions

In conclusion, despite being an exceptional encounter, gallbladder pancreatic heterotopia should be considered in the etiology of cholecystitis and as an explanation for incidental ultrasonographic findings such as gallbladder polyps, pseudo-tumoral masses, and other peculiar imaging features. In any case, the definitive diagnosis is made only by histopathological examination. Only strong cooperation between the imaging specialist, the surgical team, and the pathologist can ensure a good outcome and a proper staging of the type of pancreatic heterotopia at the level of the gallbladder. An extensive literature review concerning this entity uncovered a paucity of case reports on this matter, and the authors emphasize the importance of further data collection.

## Data Availability

Not applicable.
